# Baseline Neutrophil-to-Eosinophil Ratio Is Associated with Outcomes in Metastatic Renal Cell Carcinoma Treated with Immune Checkpoint Inhibitors

**DOI:** 10.1093/oncolo/oyac236

**Published:** 2022-11-25

**Authors:** Tony Z Zhuang, Deepak Ravindranathan, Yuan Liu, Dylan J Martini, Jacqueline T Brown, Bassel Nazha, Greta Russler, Lauren B Yantorni, Sarah Caulfield, Bradley C Carthon, Omer Kucuk, Viraj A Master, Mehmet Asim Bilen

**Affiliations:** Department of Medicine, Emory University School of Medicine, Atlanta, GA, USA; Department of Hematology and Medical Oncology, Emory University School of Medicine, Atlanta, GA, USA; Grady Cancer Center for Excellence, Grady Memorial Hospital, Atlanta, GA, USA; Departments of Biostatistics and Bioinformatics, Emory University, Atlanta, GA, USA; Department of Medicine, Massachusetts General Hospital, Boston, MA, USA; Department of Hematology and Medical Oncology, Emory University School of Medicine, Atlanta, GA, USA; Department of Hematology and Medical Oncology, Emory University School of Medicine, Atlanta, GA, USA; Grady Cancer Center for Excellence, Grady Memorial Hospital, Atlanta, GA, USA; Department of Hematology and Medical Oncology, Emory University School of Medicine, Atlanta, GA, USA; Department of Hematology and Medical Oncology, Emory University School of Medicine, Atlanta, GA, USA; Department of Hematology and Medical Oncology, Emory University School of Medicine, Atlanta, GA, USA; Department of Hematology and Medical Oncology, Emory University School of Medicine, Atlanta, GA, USA; Grady Cancer Center for Excellence, Grady Memorial Hospital, Atlanta, GA, USA; Department of Hematology and Medical Oncology, Emory University School of Medicine, Atlanta, GA, USA; Grady Cancer Center for Excellence, Grady Memorial Hospital, Atlanta, GA, USA; Department of Urology, Emory University School of Medicine, Atlanta, GA, USA; Department of Hematology and Medical Oncology, Emory University School of Medicine, Atlanta, GA, USA

**Keywords:** renal cell carcinoma, biomarker, immunotherapy, checkpoint inhibitor, nivolumab

## Abstract

**Background:**

Biomarkers have the potential to guide treatment selection and clinical care in metastatic renal cell carcinoma (mRCC) in an expanding treatment landscape. We report baseline neutrophil-to-eosinophil ratios (NER) in patients with mRCC treated with immune checkpoint inhibitors (CPIs) and their association with clinical outcomes.

**Methods:**

We conducted a retrospective review of patients with mRCC treated with CPIs at Winship Cancer Institute from 2015 to 2020 in the United States of America (USA). Demographics, disease characteristics, and laboratory data, including complete blood counts (CBC) were described at the initiation of CPIs. Clinical outcomes were measured as overall survival (OS), progression-free survival (PFS), and clinical benefit (CB) associated with baseline lab values.

**Results:**

A total of 184 patients were included with a median follow-up time of 25.4 months. Patients with baseline NER were categorized into high or low subgroups; high group was defined as NER >49.2 and low group was defined as NER <49.2 with 25% of patients in the high NER group. Univariate analyses (UVA) and multivariable analyses (MVA) identified decreased overall survival (OS) associated with elevated NER. In MVA, patients with a high baseline NER group had a hazard ratio (HR) of 1.68 (95%CI, 1.01-2.82, *P =* .048) for OS; however, there was no significant difference between groups for PFS. Clinical benefit was seen in 47.3% of patients with low baseline NER and 40% with high NER.

**Conclusions:**

We conclude that elevated baseline NER may be associated with worse clinical outcomes in mRCC. Although results require further validation, NER is a feasible biomarker in patients with CPI-treated mRCC.

## Teaser

This article reports on the role of the neutrophil-to-eosinophil ratio as a potential biomarker in metastatic renal cell carcinoma.

**Implications for Practice**

Renal cell carcinoma (RCC) is a highly immune infiltrated malignancy of which almost 20% of patients at upfront diagnosis have metastatic involvement. There is an unmet need for biomarkers to guide therapy selection in an expanding treatment landscape. We evaluate the association of clinical outcomes with baseline neutrophil-to-eosinophil ratios (NER) in patients with metastatic RCC treated with checkpoint inhibitors (CPI). Our findings indicate baseline NER may be a clinically feasible, low-cost biomarker in CPI-treated metastatic RCC with the potential to inform personalized treatment approaches.

## Introduction

Renal cell carcinoma (RCC) is within the top 10 most common malignancies worldwide. The overall incidence is approximately 16.4 per 100,000 persons with an estimated 13,000 deaths annually.^1^ Clear cell renal cell carcinoma (ccRCC) is the most common histological subtype in renal cancer, accounting for approximately 80% of RCC cases.^2^ IMDC (International Metastatic RCC Database Consortium) score is a prognostic tool that predicts survival in patients with metastatic RCC, originally validated for patients receiving vascular endothelial growth factor receptor inhibitors (VEGFRi). Around 16% of patients with renal cancer have metastatic involvement upon initial diagnosis with a recurrence rate of 30% and a 5-year survival rate of approximately 14%, highlighting the importance of disease risk stratification and treatment selection in a growing treatment landscape.^3^

ccRCC is a heterogenous, highly immune infiltrated malignancy that demonstrates a robust response to immune checkpoint inhibitors (CPIs).^4-8^ Upfront therapy consists of a dual ICI or ICI-VEGFRi. First-line regimens include nivolumab and cabozantinib, pembrolizumab and lenvatinib, pembrolizumab and axitinib, or axitinib and avelumab for all risk groups while nivolumab and ipilimumab are typically reserved for intermediate to poor-risk disease.^9-17^ CheckMate-214, JAVELIN-101, Keynote-426, CheckMate-9ER, and CLEAR are some of the landmark studies examining clinical outcomes, ultimately leading to FDA approval of first-line dual ICI and ICI/VEGFRi-based treatments.^18-21^

Neutrophils, T cells, and macrophages play key roles in inhibiting tumorigenesis in the microenvironment. The ­neutrophil-to-lymphocyte ratio (NLR) has served as a surrogate for assessing disease severity and response to therapy in mRCC.^22,23^ Several studies in melanoma and endometrial cancer demonstrate an elevated relative eosinophil count may also confer a better response to upfront immunotherapy.^24,25^ However, this has not been fully evaluated in mRCC. To investigate the role of neutrophil-to-eosinophil ratio (NER) as a potential biomarker in mRCC, we studied associations with overall survival (OS), progression-free survival (PFS), and overall clinical benefit (CB) upon CPI initiation.

## Methods

### Patients and Data

Patient records with a diagnosis of mRCC were compiled from a database at the Winship Cancer Institute of Emory University from 2015 to 2020 in the United States of America (USA). Patients were treated with various combinations of CPI consisting of nivolumab and ipilimumab, pembrolizumab, or avelumab. Institutional Review Board approval was obtained. Data collected included demographics, treatments, outcomes, and labs which included mainly a baseline complete blood count (CBC). We calculated NER based on the CBC and those measurements were dichotomized as high vs low subgroups.

### Definitions

Patients were prognosticated based on IMDC criteria. Normal lab ranges included absolute neutrophil count (0.91-5.53 × 10^3^ cells/µL) and absolute eosinophil count (≤0.36 × 10^3^ cells/µL). Clinical outcomes included OS calculated from the time of CPI initiation to death. PFS was calculated from CPI initiation to clinical progression, radiographic progression based on RECIST 1.1 criteria or death, whichever event occurred first. Clinical benefit was defined as achieving complete response (CR), partial response (PR), or stable disease (SD). Objective response rate (ORR) was defined as the percentage of patients with CR and PR after receiving 3 cycles of CPI. Patient deaths were confirmed by reviewing the electronic health record as well as the Georgia state obituary database.

### Statistical Analysis

Statistical analysis was conducted using SAS version 9.4 and SAS macros.^26^ The significance level was set at *P* < .05. Descriptive statistics for each variable were reported. The univariate associations (UVA) and multivariable analyses (MVA) for OS or PFS were tested by Cox proportional hazard model with hazard ratios (HR) and its 95% CI being reported. Variables controlled in the MVA were gender, race, IMDC, histology, and prior lines of therapy. The cutoff value of NER was determined by first examining the nonlinear relationship between numerical NER and PFS/OS and then categorizing it into 4 quartiles. High NER was set as the top quartile value with a value >49.2.

## Results

### Overview of Results

A total of 184 patients with metastatic disease were included with a median follow-up time of 25.4 months (Table 1). The median age was 63 years (23-90 years). Approximately 72% of patients were male, 20% were Black, and 17% had ECOG performance scores ≥2. Approximately 78% of patients had ccRCC histology and 40% were treatment-naïve. Of those who received prior treatment (*n* = 110), nearly all patients (95.5%) received VEGF-based therapy. Fifty-two patients (28%) had liver metastases. Per IMDC criteria, 18%, 56%, and 26% of patients were favorable, intermediate, and poor risk respectively. The median baseline NER was 25.5 (2.1-405). There were no significant differences in baseline characteristics such as age, gender, race, BMI, and ECOG/PS between high- and low-baseline NER groups. There was a significantly greater proportion of patients with poor-risk IMDC in the high baseline NER group compared to those in the low NER group (19.7% vs 45.7%). Median OS and PFS for the overall cohort were 25.0 months and 4.3 months respectively. Similar ORR were seen in 24.4% of patients with low baseline NER and 21.9% in high baseline NER groups (Table 2). Clinical benefit was seen in 47.3% of patients with low baseline NER and 40% with high NER.

**Table 1.** Baseline characteristics of patients with mRCC treated with CPI.

Variables of interest	Values
Age, years, median (range)	63 (23-90)
Gender, *n* (%)	
Male	132 (71.7)
Female	53 (28.3)
ECOG performance status^a^, *n* (%)	
0	64 (35.6)
1	86 (47.8)
≥2	30 (16.7)
Clear cell histology, *n* (%)	139 (78.5)
IMDC risk group^b^, *n* (%)	
Favorable	33 (18.0)
Intermediate	102 (55.7)
Poor	48 (26.2)
Number of distant metastases, *n* (%)	
1	29 (15.8)
2	68 (37.0)
3	87 (47.3)
Liver metastases, *n* (%)	52 (28.3)
Prior treatment (VEGF inhibitor-based) , *n* (%)	
Yes	106 (95.5)
No	4 (4.5)
Prior lines, *n* (%)	
0	74 (40.2)
1	78 (42.4)
≥2	32 (17.4)
Baseline NER (2-level)^c^, *n* (%)	
Low	138 (75)
High	46 (25)

^a^Eastern Cooperative Oncology Group classification ranging from 1 to 5, with lower scores indicating better functionality.

^b^International Metastatic RCC Database Consortium used to risk-stratify disease within favorable, intermediate, and poor risk classes.

^c^Optimal cut: NER >49.2 with 25% of patients in the high NER subgroup.

Abbreviations: mRCC, metastatic renal cell carcinoma; CPI, checkpoint inhibitors; ECOG, Eastern Cooperative Oncology Group; IMDC, International Metastatic RCC Database Consortium; NER, ­neutrophil-to-eosinophil ratios.

**Table 2.** Stratified covariates based on low vs high baseline NER.

Covariate	Subclasses	Low baseline NER (*n* = 138)^c^	High baseline NER (*n* = 46)	*P*-value^d^
Clinical benefit^a^	Y	62 (47.3)	16 (40)	.415
	N	69 (52.7)	24 (60)	
Objective response^b^	Y	32 (24.4)	9 (21.9)	.745
	N	99 (75.6)	32 (78.1)	
Best response	CR	8 (5.8)	2 (4.3)	.724
	NE	7 (5.1)	5 (10.9)	
	PD	57 (41.3)	19 (41.3)	
	PR	24 (17.4)	7 (15.2)	
	SD	42 (30.4)	13 (28.3)	
Median baseline NER	Median (min-max)	19.56 (2.14-49.15)	68 (49.2-405)	<.001*
Median age (years)	Median (min-max)	62 (23-90)	64.5 (41-85)	.160
Baseline BMI, kg/m^2^	<25	47 (34.3)	19 (41.3)	.392
	≥25	90 (65.7)	27 (58.7)	
Gender	M	99 (71.7)	33 (71.7)	1.000
	F	39 (28.3)	13 (28.3)	
Race	Black	24 (17.4)	12 (26.1)	.198
	Non-black	114 (82.6)	34 (73.9)	
Histology	ccRCC	104 (78.8)	35 (77.8)	.887
	Others	28 (21.2)	10 (22.2)	
ECOG performance status	0-1	115 (85.2)	35 (77.8)	.275
	≥2	20 (14.8)	10 (22.2)	
Number of distant metastases	1	23 (16.7)	6 (13.0)	.063
	≥2	115 (83.3)	40 (87.0)	
Presence of liver metastases	Yes	36 (26.1)	16 (34.8)	.257
	No	102 (73.9)	30 (65.2)	
Prior number of treatment lines	0	51 (37.0)	23 (50.0)	.241
	≥1	87 (63.0)	23 (50.0)	
IMDC risk class	Favorable	22 (16.1)	11 (23.9)	<.001*
	Intermediate	88 (64.2)	14 (30.4)	
	Poor	27 (19.7)	21 (45.7)	

Boldened *P*-values indicate statistical significance. Asterisks also indicate statistical significance.

^a^Clinical benefit defined as complete response (CR), partial response (PR), or stable disease (SD).

^b^Objective response defined as complete response (CR) or partial response (PR).

^c^An optimal cut of 49.2 was used to divide baseline NER into low and high subclasses.

^d^The *P*-value is calculated by ANOVA for numerical covariates and chi-square test or Fisher’s exact for categorical covariates, where appropriate.

Abbreviations: NER, neutrophil-to-eosinophil ratios; CR, complete response; NE, non-evaluable disease; PD, [NEED DEFINITION]; PR, partial response; SD, stable disease; BMI, body mass index; M, male; F, female; ccRCC, clear cell renal cell carcinoma; ECOG, Eastern Cooperative Oncology Group; IMDC, International Metastatic RCC Database Consortium.

### Overall Survival for Neutrophil-to-Eosinophil Ratio

**Figure 1.**
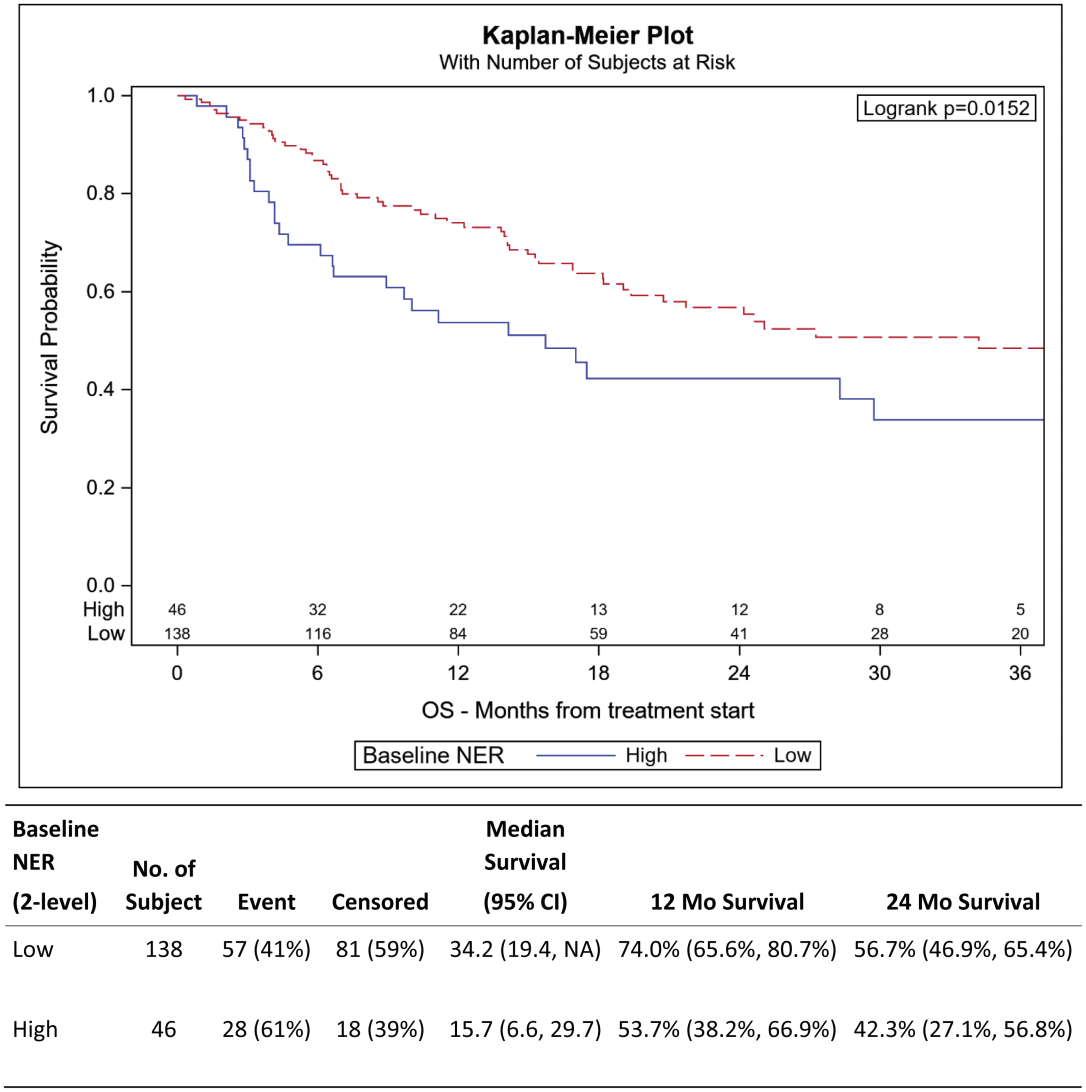
Kaplan–Meier plot for OS. Baseline NER at optimal cut. OS was significantly associated with NER.

Elevated NER was associated with shorter OS. Patients with baseline NER were categorized into high or low subgroups; high defined as NER >49.2 and low defined as NER <49.2 with 25% of patients in high NER group. UVA and MVA identified worse clinical outcomes (CO) associated with elevated NER. UVA for OS demonstrated decreased survival in patients with elevated NER with HR 1.74 (95% CI, 1.11-2.74, *P* = .017). After controlling for gender, race, IMDC risk score, histology, and prior lines of therapy in MVA, patients with high baseline NER group had an HR of 1.68 (95% CI, 1.01-2.82, *P* = .048) for OS (Table 3). The 12-month survival rate was 74% among low NER group and 53.7% among high NER group (Fig. 1).

**Table 3.** Tabulated OS and PFS by baseline NER at optimal cut.

	PFS				OS			
	UVA		MVA^a^		UVA		MVA	
	HR	*P*-value	HR	*P*-value^b^	HR	*P*-value	HR	*P*-value
Low NER	1.23 (95%CI, 0.85-1.78)	.265	1.37 (95%CI: 0.92-2.04)	.119	1.74 (95%CI: 1.11-2.74)	**.017**	1.68 (95% CI, 1.01-2.82)	**.048**
High NER	Ref	–	Ref	–	Ref	–	Ref	–

^a^The MVA was built by controlling for gender, race, IMDC risk score, histology, and prior lines of therapy.

^b^Statistical significance at alpha <0.05.

### Progression-Free Survival for ­Neutrophil-to-Eosinophil Ratio

**Figure 2.**
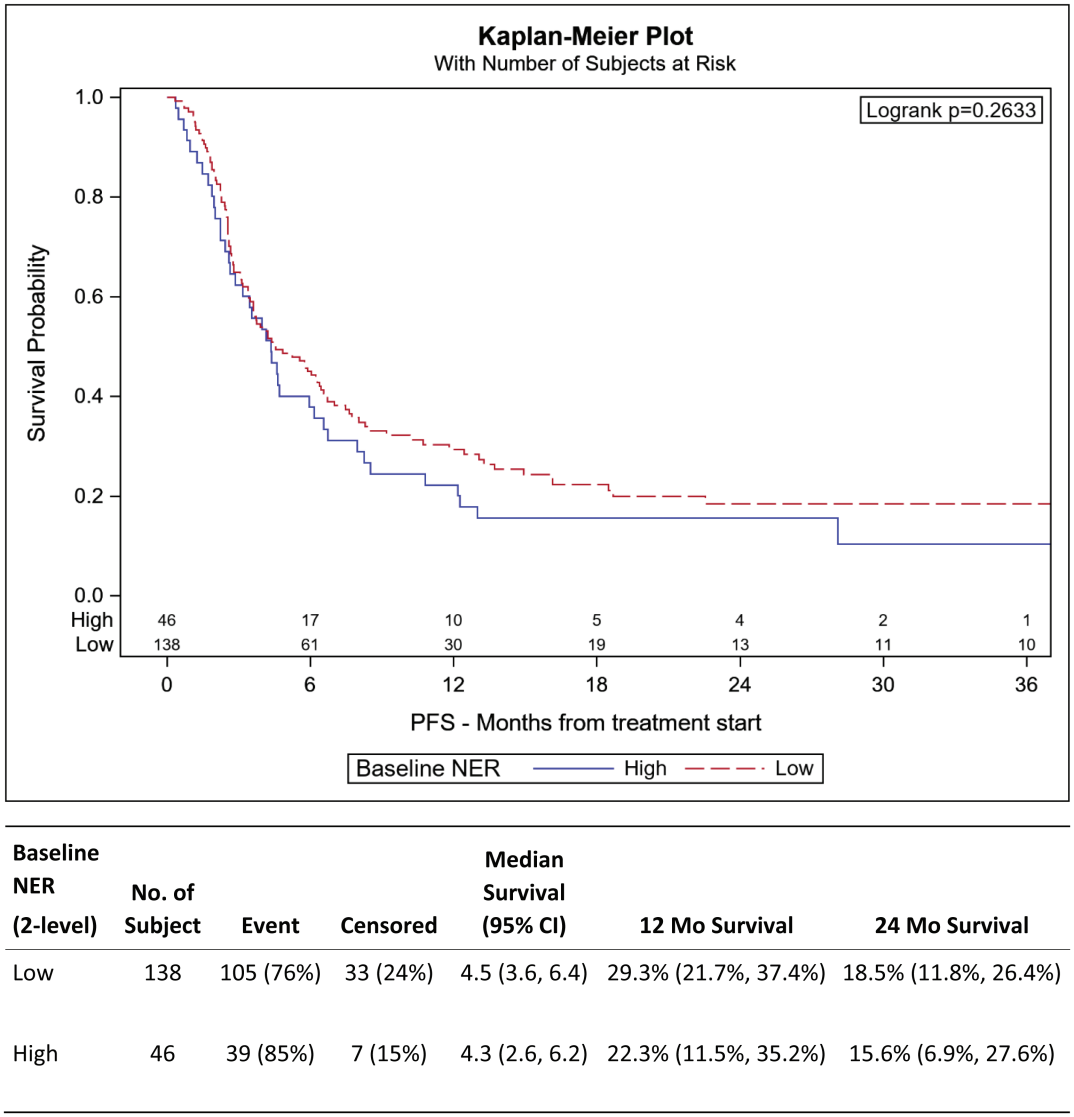
Kaplan–Meier plot for PFS. Baseline NER at optimal cut. PFS was not significantly associated with NER.

UVA and MVA for PFS were not significantly associated with elevated NER. UVA at optimal cutoff demonstrated HR 1.23 (95% CI, 0.85-1.78, *P* = .265) while MVA at optimal cutoff with HR 1.37 (95% CI, 0.92-2.04, *P* = .119) (Table 3). The 12-month progression-free rate was 29.3% among low NER group and 22.3% among high NER group (Fig. 2).

## Discussion

Our study evaluated the feasibility of baseline NER as a biomarker to forecast outcomes in metastatic RCC upon initiation of CPI therapy. This analysis demonstrates an association of elevated baseline NER with worsened survival outcomes at the start of immunotherapy. After adjustment for gender, race, IMDC risk score, histology, and number of prior lines, patients with lower baseline NER had a 1.7-fold increase in overall survival. Thus, baseline NER may be a feasible low-cost, clinical laboratory-based biomarker to prognosticate patient outcomes after CPI treatment.

An expanding treatment landscape in mRCC creates an unmet need to identify biomarkers of clinical outcomes with immunotherapy. The tumor microenvironment involves a complex interplay of immunoregulatory activity between tumor cells and white blood cells. Lymphocytes are postulated to increase anti-tumor response with enhanced activity upon initiation of CPI therapy. Thus, decreased lymphocytes may confer a less robust response to immunotherapy, resulting in less immune-mediated control within the tumor microenvironment.

Neutrophil-to-lymphocyte (NLR), monocyte-to-­lymphocyte (MLR), and platelet-to-lymphocyte (PLR) ratios, CRP, and skeletal muscle indices are the most studied biomarkers in genitourinary malignancies.^16,23,27,28^ A phase I clinical trial demonstrated an association of worsened clinical outcomes with elevated NLR, MLR, and PLR levels in advanced carcinomas.^29^ In fact, NLR has become increasingly used as a marker of response upon CPI initiation as another investigation revealed an association of improved clinical outcomes with lower baseline NLRs in mRCC.^30^ A sub-analysis in the JAVELIN Renal 101 trial demonstrated that patients with lower baseline NLR at the start of CPI and TKI therapy had longer observed OS, ORR, and PFS than those with higher levels.^23^ In addition to NLR, patients with elevated MLR and PLR levels have also been found to have worsened survival outcomes in the renal, bladder, and urothelial carcinomas.

Although the role of eosinophils is more familiar in the context of atopic, parasitic, and viral diseases, its role in the tumor microenvironment is less as well understood compared to its other leukocyte subtypes.^31^ Numerous studies have demonstrated the integral role of eosinophils in disease progression or metastasis within the tumor microenvironment. Several in vitro studies postulate eosinophils mediate tumor destruction via crosstalk with B-cells, Th1 and Th2 CD4+ T cells, and granulocytes. Through recognition of distinct tumor-associated molecular markers as well as facilitation by other leukocytes, eosinophils degranulate and subsequently release TNF-α, granzymes, major essential protein (MBP), and metalloproteinases with a wide catch-net of effects involving recruitment of other leukocytes, antigen presentation to T cells, and tumor cell destruction.^32^ The release of ribonucleases and cationic proteins forms a cytotoxic extracellular trap driving tumor cell death. In vivo studies suggest CC-chemokine ligands promote both eosinophil recruitment and subsequent tumor destruction in malignancies. CCL5, CCL-11, CXCL9, and CXCL10 are postulated as the main drivers in eosinophil-mediated tumor cell necrosis. Notably, decreased CCL-11 expression is associated with increased tumor burden and absence of eosinophils compared to ­CCL-11-rich involvement in murine models.^33^ Previous in vitro studies demonstrate tumor cell death recruits eosinophils within melanoma models. Although the biological mechanism is unclear, retrospective clinical studies in melanoma reveal a potential association of lower baseline median NER and eosinophil counts with improved outcomes at upfront immunotherapy.^34^

Our work demonstrates concordant outcomes and disease trajectories in metastatic RCC compared to prior studies with similar OS and clinical benefits. A post hoc analysis of the JAVELIN 101 study, exploring the clinical benefit of avelumab and axitinib, revealed an association of lower NER with better PFS and ORR. A retrospective, ­multi-center study consisting of 162 patients with mRCC exhibited similar survival findings.^35^ A follow-up study at ASCO 2022 performed a landmark analysis of NER changes at week 6 of nivolumab/ipilimumab. It showed clinical benefit with 68% of patients having decreased NER at week 6. Decreased NER ≥50% was associated with longer OS [adjusted HR (AHR) 0.38 (0.17-0.85), *P*-value .02] and PFS [AHR 0.55 (0.31-0.95, *P*-value .03)]. These findings were preserved on stratified analysis with high baseline NER.^36^ In addition, a retrospective analysis demonstrated a potential association of elevated absolute eosinophil count with increased immune-related adverse events upon CPI treatment.^37^ As a result, NER may serve as a clinically useful and feasible biomarker in assessing patient outcomes with CPI treatment and disease risk stratification within an expanding mRCC treatment landscape.

Tucker et al also explored clinical outcomes associated with NLR upon initiation of CPI initiation, demonstrating longer OS in those with NLR below the median cutoff in comparison.^35^ As opposed to NER, there were no differences in ORR or PFS between the 2 NLR groups for patients with intermediate/poor-risk mRCC. While NLR has been validated as a biomarker of response in mRCC treated with VEGFR tyrosine kinase inhibitors and other forms of therapy, NER may be more prognostic of disease trajectories in mRCC treated with immunotherapy. More prospective and larger studies are needed to further explore NER’s role as a viable biomarker.

However, there are some limitations to our study. First, this investigation was a retrospective study and is largely descriptive. Neutrophils and eosinophils may be affected by infections and other comorbidities. These are potential confounding factors that were not accounted for. Furthermore, most of the patients in this cohort were of the white race and thus the analysis did not account for a more diverse population. We included data on patients treated with other forms of therapy such as tyrosine kinase inhibitors or VEGFRi of which treatment outcomes may serve as a potential avenue for further validation of NER as a prognostic tool. An understanding of eosinophils in the mRCC immune-tumor microenvironment may be furthered with the validation of dynamic changes in NER with histopathologic data in chemokine expression (eg, CCL, CXCL).

## Conclusion

Elevated baseline NER may be associated with shorter OS with mRCC treated with the initiation of CPI. To our knowledge, our investigation is one of the most comprehensive studies to identify baseline NER as a feasible prognostic low-cost laboratory-based biomarker. This retrospective study is hypothesis-generating. Larger prospective data are needed for further validation of NER as a biomarker.

## Data Availability

The data underlying this article will be shared on reasonable request to the corresponding author.
